# Assessment and improvement of image homogeneity in black-blood T2-weighted turbo spin-echo CMR

**DOI:** 10.1186/1532-429X-14-S1-O113

**Published:** 2012-02-01

**Authors:** Benjamin Wince, Lowie M Van Assche, Han W Kim, Lubna Bhatti, Christoph J Jensen, Elizabeth Jenista, Wolfgang G Rehwald, Deneen Spatz, Yong-Yin Kim, Michele Parker, Raymond J Kim

**Affiliations:** 1 Cardiology, Duke University, Durham, NC, USA; 2 Siemens Medical Systems R&D, Chicago, IL, USA

## Background

Double inversion recovery (DIR) and triple inversion recovery (TIR) prepared Turbo-Spin-Echo (TSE) are the most commonly used sequences for black-blood T2-weighted (T2W) cardiac magnetic resonance. For both, cardiac motion often leads to signal loss and image inhomogeneity, which can affect diagnosis. Signal loss artifacts are thought to be due to misalignment of the black-blood preparation with readout. However, the TSE readout itself is also motion sensitive and could lead to signal loss. We examined image homogeneity in routine Black-Blood T2W-TSE and investigated potential improvement by reducing interecho-spacing of the TSE readout.

## Methods

Ten healthy volunteers underwent T2W-CMR using 3 sequences: (a) Standard DIR-TSE (interecho-spacing = 9.66ms), (b) Standard TIR-TSE (interecho-spacing = 9.66ms) and, (c) a Modified DIR-TSE sequence with low interecho-spacing (interecho-spacing = 3.48ms). Reduction in interecho-spacing was accomplished by increasing bandwidth >4-fold and utilizing shorter refocusing pulses. Total readout time was held constant for all three sequences, leading to shorter breath-hold times for the modified sequence. In each volunteer, a mid-ventricular short-axis slice was repeatedly imaged with only the timing of readout in diastole changed by 50 ms increments (between 60-100% of the cardiac cycle). All images were acquired using coil normalization, slice thickness=7mm, and effective echo-time=60ms. Epicardial and endocardial contours were planimetered. Mean image intensity and standard deviation (SD) were measured for the slice. Image homogeneity was defined as the ratio of SD/mean image intensities x100 (higher values indicate worse homogeneity). The sensitivity of each sequence to readout timing was determined by the maximum change in image homogeneity across the different timepoints of diastole.

## Results

**Figure 1:**
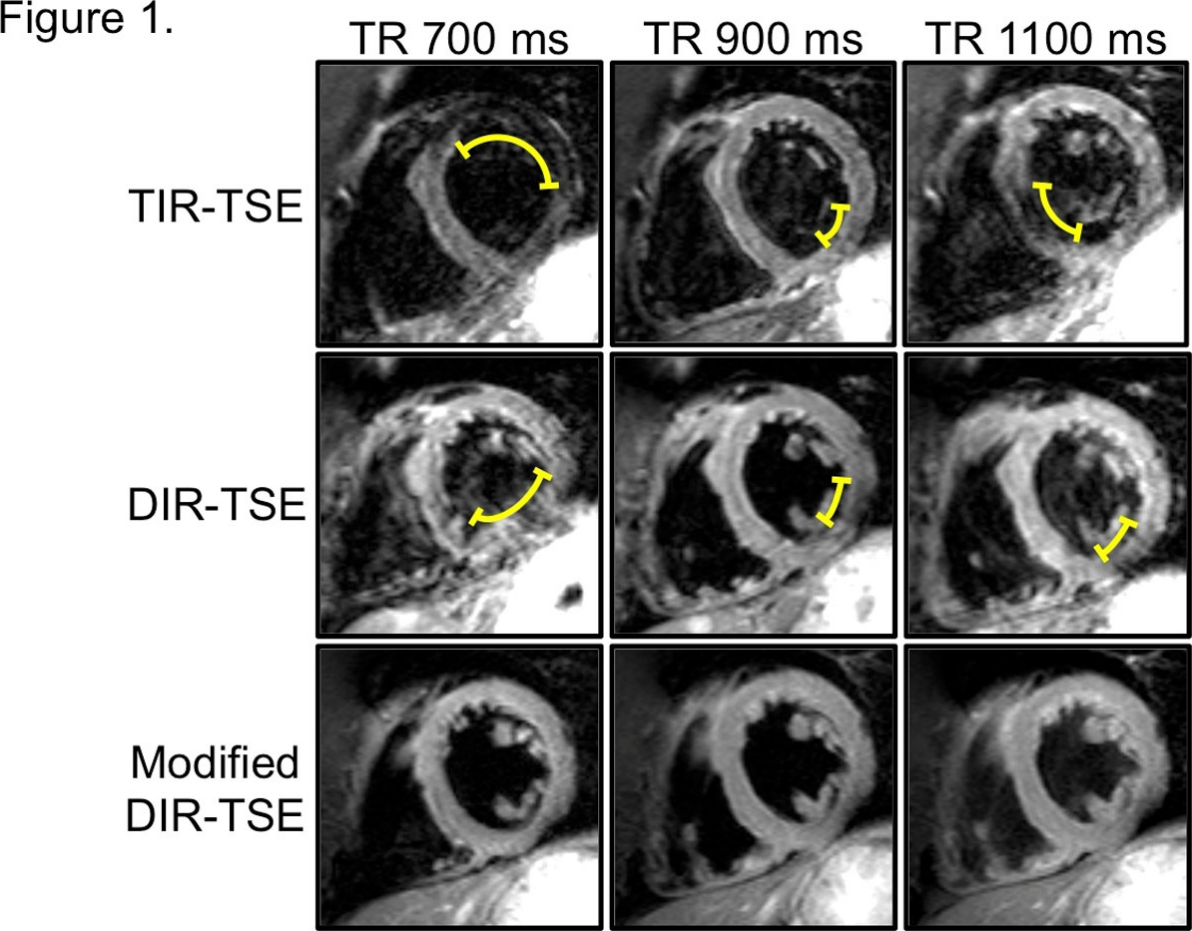


Overall, mean image homogeneity was best for Modified DIR-TSE (16.1±7.9), compared with Standard DIR-TSE (23.5±11.3) and Standard TIR-TSE (27.1±13.9, both p<0.03). Modified DIR-TSE was also less sensitive to readout timing, as the maximum change in image homogeneity across diastole was smaller at 14.4±9.5 versus 23.2±15.9 and 33.6±11.1 for Standard DIR-TSE and TIR-TSE, respectively (p<0.05 for both comparisons). Figure 1 shows representative images demonstrating improved image homogeneity for the Modified DIR-TSE sequence as well as showing less sensitivity to changes in the timing of readout.

## Conclusions

T2-weighted-CMR with Standard-DIR and TIR-TSE is limited by image inhomogeneity and is sensitive to small changes in readout timing. A Modified DIR-TSE with reduced interecho-spacing of readout significantly improves image homogeneity and robustness to variation in readout timing. This may reduce the risk of misdiagnosis.

## Funding

Funded in part by 5R01HL064726-07.

